# Prolonging the Lifetime of Wireless Sensor Networks Interconnected to Fixed Network Using Hierarchical Energy Tree Based Routing Algorithm

**DOI:** 10.1155/2014/158420

**Published:** 2014-10-27

**Authors:** M. Kalpana, R. Dhanalakshmi, P. Parthiban

**Affiliations:** ^1^Department of Communication Systems, Sethu Institute of Technology, Virudhunagar 626 115, India; ^2^HCL Technologies, Chennai, India; ^3^National Institute of Technology, Tiruchirappalli, India

## Abstract

This research work proposes a mathematical model for the lifetime of wireless sensor networks (WSN). It also proposes an energy efficient routing algorithm for WSN called hierarchical energy tree based routing algorithm (HETRA) based on hierarchical energy tree constructed using the available energy in each node. The energy efficiency is further augmented by reducing the packet drops using exponential congestion control algorithm (TCP/EXP). The algorithms are evaluated in WSNs interconnected to fixed network with seven distribution patterns, simulated in ns2 and compared with the existing algorithms based on the parameters such as number of data packets, throughput, network lifetime, and data packets average network lifetime product. Evaluation and simulation results show that the combination of HETRA and TCP/EXP maximizes longer network lifetime in all the patterns. The lifetime of the network with HETRA algorithm has increased approximately 3.2 times that of the network implemented with AODV.

## 1. Introduction

Most of the wireless sensor networks (WSN) cannot be operated in isolation. They must be connected with fixed network for monitoring and controlling. Wireless sensor network uses many small sensor nodes for collecting information. These physically small and inexpensive nodes are equipped with one or more sensors, low range RF transceiver, an embedded controller, and a battery as power source. These sensor nodes are deployed in large numbers, hundreds to thousands to form a network. These nodes cannot be configured manually due to its large number and hence they need to configure themselves. In many situations these nodes are not accessible for replacing batteries. Hence, efficient utilization of energy is very essential. Most of the sensor network applications are aimed at monitoring or detecting of physical phenomena. Examples include office building environment control, wild-life habitat monitoring, smart spaces, medical systems, robotic exploration, alarm systems, forest fire detection, and monitoring of volcanic eruptions. For such applications, the sensor networks cannot operate in complete isolation; they must be connected to fixed network [1, 2]. By connecting the sensor network to fixed network infrastructure such as the global internet, remote access to the sensor network is possible. By using TCP/IP protocol which is the de facto standard for network, the sensor network is directly connected to the fixed network (wired TCP/IP network) through gateway. The most important consideration for a WSN is the energy consumption [3]. Though the WSN seems to be applicable in variety of systems, widespread adoption of WSN will not be possible if the batteries needed to be changed constantly. Therefore, sensor node is to be designed to minimize energy consumption. The energy could further be managed efficiently by providing a better route and minimizing the number of packet drops [4–8]. The efficiency also depends on how the nodes are placed [9].

This paper proposes a routing algorithm, a congestion control algorithm, and seven fixed node distribution (deployment) patterns with the aim of improving the energy efficiency of the WSNs. The proposed routing algorithm is named as hierarchical energy tree based routing algorithm. The routing of packets to home node in this algorithm is done more efficiently based on the remaining energy in each node. A logical hierarchical energy tree (HET) is formed based on the remaining energy in each node and the nodes are placed at level 1 or level 2. The destination node is the root node. The directional characteristics (unidirectional or bidirectional) are fixed based on the position of the nodes (level 1 or level 2) and routing is done accordingly. This avoids the unnecessary communications between neighbors which leads to energy saving. Energy information from neighboring nodes is obtained by transmitting a one hop beacon request. The energy information is used to form the HET and routing decision is made using the neighboring information and position of node in the HET. Bidirectional or unidirectional transmissions of packets are done based on this information stored on the logical HET.

The phenomenon of congestion can be observed in different types of wired and wireless networks even in the presence of robust routing algorithms. Frequent data collection is required in applications like environment monitoring and habitat monitoring. These data collected periodically and were sent to data center or sink node [10, 11]. It is known that each sensor node can only be equipped with a limited amount of storage. Hence, congestion builds up if at any given routing node the data collection rate is more than that of the data forwarding rate. This type of congestion phenomenon that occurs because of many-to-one transmission is known as funneling effect. This paper proposes a congestion control algorithm based on the modified AIMD rule for increasing and decreasing the congestion window. The modification is done by exponentially increasing and decreasing the window size. This algorithm is named as TCP/EXP. The lifetime of the sensor network is another important parameter. Lifetime of a WSN can be defined as the time interval between the deployment of the sensor field and the time when the network breaks. Congestion severely affects the performance of a wireless sensor network in two aspects: increased data loss and reduced lifetime. Hence it is also essential to control congestion in wireless sensor networks. 

The two proposed algorithms are implemented in ns 2 with seven node distribution patterns. The performance of these algorithms is evaluated and compared with the existing routing algorithms and congestion control algorithms. The paper is organized as follows: Section 2 discusses the routing algorithm HETRA.  Section 3 discusses the exponential congestion control algorithm (TCP/EXP).  Section 4 discusses an NLT model used for evaluation.  Section 5 discusses the simulation of node distribution patterns. The results are also analyzed in Section 6. The last section concludes the analysis.

## 2. Hierarchical Energy Tree Based Routing Algorithm (HETRA)

Energy remaining in sensor nodes is used as additional parameter for taking routing decision in the proposed HETRA. The remaining energy in the sensor nodes is used to place the nodes in either Level 1 or Level 2 in the logical HET generated. Nodes at Level 1 or Level 2 are categorized as bidirectional or unidirectional. Periodic update of HET is made by collecting information from neighboring nodes using beacon request packet.

**Figure 1:**
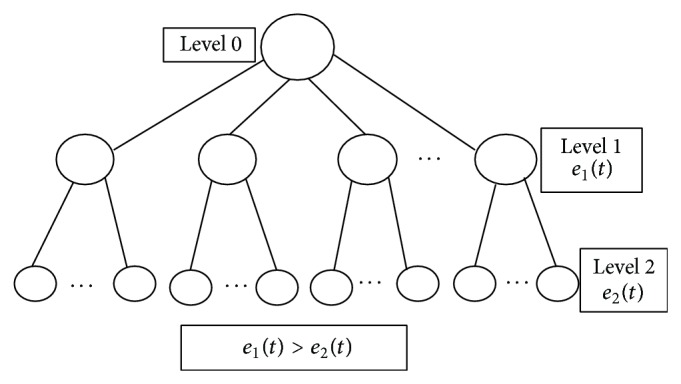
Hierarchical energy tree.

HET is constructed by comparing the remaining energy in each sensor nodes. HET is a three-level logical tree. The data center destination node is the Level 0 node. Energy information collected from the neighbor sensor node is compared with a threshold energy level for placing the nodes in Level 1 or Level 2. Nodes with energy more than the threshold energy are placed at Level 1 and the remaining nodes with energy less than the threshold energy are placed at Level 2 as shown in Figure 1.

**Figure 2:**
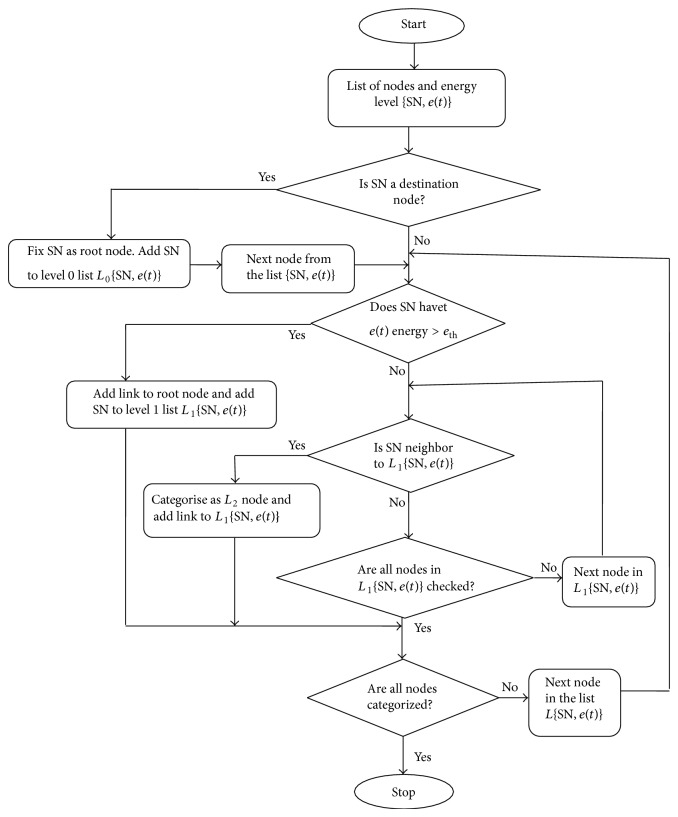
HET construction flowchart.

Level 1 nodes, having higher energy, are categorized to have bidirectional links. Level 2 nodes are categorized to have unidirectional links and will only receive the information collected from the neighbor sensor nodes. Copy of the HET constructed as above is sent to other nodes for taking routing decision along with the other routing metrics. The HET construction algorithm and flowchart are given in Algorithm 1 and Figure 2.

For constructing/updating HET, sensor nodes with more energy (Level 1) will broadcast a beacon request to all the neighboring sensor nodes to collect the information. The neighboring Level 1 nodes will in turn will respond these beacon requests be sending the remaining energy information, location information, and so forth. When HET is used for routing/forwarding the information from the sensor nodes to the data center destination node, the sensor node will always select the neighboring Level 1 node that is the closest to the destination node. Routing is changed when the forwarded packet reaches a node that has no Level 1 neighboring node closest to the destination node.

HETRA is implemented in ns 2. As the nodes involved in transmission and reception are decided based on the energy, the nodes are effectively managed by controlling the energy consumption. If the energy of the nodes is less, then they are not involved in transmission and hence the overall lifetime of the node is increased and consequently the network lifetime is increased. Hence with the same energy the network is able to sense and transmits the sensed data a long time. This is equivalent to network having more energy or network utilizing less energy for transmission and reception. This shows that HETRA is more energy efficient.

## 3. Exponential Congestion Control Algorithm (TCP/EXP)

This section discusses the main properties of the proposed congestion control algorithm based on exponential distribution. The proposed algorithm makes use of the window adjustment mechanism in addition to congestion detection, packet retransmission, and round trip time estimation, and so forth, used in the TCP algorithm. The main objective of the proposed exponential congestion control algorithm is to increase the window size faster and thus acquire more bandwidth quicker.

Congestion direction is usually very difficult. This is because it is not easy to determine the status of network. Whenever the number of data packets to be transmitted is very high (as in the case of burst traffic), the nodes are unable to transmit all the packets at the same rate as they are received. Also, nodes cannot store all the received packets due to the limitation of buffer size. This leads to congestion of data packets at the nodes. Nowadays the availability of bandwidth increases rapidly. Different types of software nowadays necessitate the need for improving the utility of this increased bandwidth by modifying the TCP congestion control mechanism [12].

As a connection-oriented protocol, TCP provides a reliable data transfer along with flow and congestion control. The number of outstanding data packets that remain to be acknowledged is maintained by the TCP congestion control mechanism [13]. During start-up, TCP have a slow-start mechanism that increase the data rate twice for every round trip time. When a packet is dropped or lost, congestion window size is reduced to 1 and TCP enters the slow-start phase again. Packet loss is indicated by the receipt of three consecutive duplicate acknowledgements. The increase and decrease rules of the AIMD algorithm [14, 15] are modified such that the window size is controlled by using an exponential of the previous window size. These rules form the basis for the exponential congestion control algorithms.

The increase and decrease rules of TCP/EXP algorithm are given below:

(1) $$\begin{array}{c} I:W_{t+R}⟵W_{t}+{\left(\frac{\alpha }{e^{Wt}}\right)}^{k},\\ D:W_{t+\delta t}⟵W_{t}-{\left(\beta e^{Wt}\right)}^{l}, \end{array}$$

where *I* → increase in window size, *D* → decrease in window size, *W*_*t*_ → window size at time *t*, *R* → round trip time of the flow, *α* and *β* → increase and decrease rule constants, and *K* and *l* → increase and decrease rule exponent.

The above two rules indicate the way in which the window size is increased and decreased exponentially. This exponential increase is mainly to acquire more bandwidth by way of exponentially varying the window size in the increase rule. Similarly whenever the packet drops, the window size is exponentially decreased as per the decrease rule. A set of exponential rules are formed by choosing different values of *α* and *β* in (1). If higher order terms are also included in (1), exponential algorithms of different orders will be available. These may be used for the window size adjustment for increase and decrease. This work assumes one set of values chosen for *α*, *β*, *k*, and *l*. The values of these parameters are chosen after the algorithm is implemented in ns 2. A number of simulations are performed by varying the values of *α*, *β*, *k*, and *l*. Among these an optimal set of values that provide a higher bandwidth with minimal packet drops is chosen. With these specific values the algorithm is used for the proposed WSN, along with the proposed routing algorithm HETRA. AIMD is suitable for traffic where the data is transferred at almost fixed rate and unsuitable for burst traffic. The exponential congestion control algorithm has the property of increasing the window size as burst and hence more suited for burst traffic as in video streaming applications.

## 4. Modeling Network Lifetime of Wireless Sensor Network

Modeling and analysis of NLT of WSN is proposed by a number of researchers. Their work chose a generic model for the NLT. NLT model is proposed for a cluster based WSN and the researchers have also proposed a model for NLT in order to maximize the NLT by placing the nodes in the optimal positions [16, 17]. Similarly a number of other researchers suggest a model specific to the environment and application for which WSN is intended [18–20]. There is no generalized approach available to be used for any type of network. Most of the researchers used heuristic approach for modeling the system.

This paper proposes a model for NLT developed by heuristic and on the concept used by the researchers [16–20]. The maximum time for which the network is alive is found from the values of initial energy and the energy consumed for the transmission, reception, and processing of the sensed data by various sensor nodes.

In general, the maximum network lifetime (NLT_max⁡_) may be defined as

(2) $$\begin{array}{c} {\text{NLT}}_{\max}=\frac{E_{i}}{P_{T}}, \end{array}$$

where *E*_*i*_ is the initial energy of the sensor node which is represented by Joules or watt seconds and *P*_*T*_ is the total energy consumed by the sensor node for sensing and transmitting the sensed data to the base station node which is represented by watt. The value of *P*_*T*_ may be evaluated as

(3) $$\begin{array}{c} P_{T}=\left(e_{t}+e_{r}+e_{p}\right)\times f\left(x\right)\times n_{\text{avg}}, \end{array}$$

where *e*_*t*_ is the power consumed for transmitting the data, *e*_*r*_ is the power consumed for receiving the data, *e*_*p*_ is the power consumed for sensing and processing the data, *f*(*x*) is the weighting function, and *n*_avg_ is the average number of nodes involved in the transmission of data from the source to the base station.

**Figure 3:**
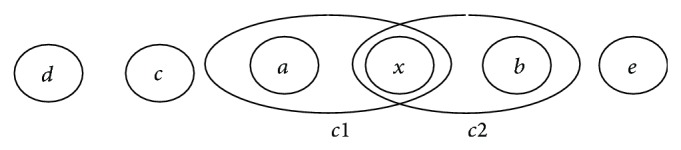
Node “*a*” or “*b*” senses the data and transmits to the base station “*x*.”

The total power consumed (*P*_*T*_) is given by power consumed by a node for sensing and processing, transmitting the packet to the neighbor nodes and receiving the packets by the intermediate nodes. There are a number of intermediate nodes involved for forwarding the sensed data packet towards the base station. The total power (*P*_*T*_) should include the power consumed by all these nodes. Hence, the power consumed is multiplied by the average number of nodes involved for transmission of sensed data to the base station node. In a wireless sensor network, all the nodes are distributed over the sensing field based on a particular topology or pattern. The phenomenon to be sensed could occur at any place randomly. This phenomenon will be sensed by the node closer to the occurrence of the phenomenon. The node sensing the phenomenon could be a node closer to the base station node. Hence, the number of nodes involved in transmitting the sensed data may vary. But the minimum number of nodes involved in a transmission should be 2 as shown in Figure 3.

As shown in the figure, the node “*a*” senses the data and transmits it to the base station node “*x*,” which is the neighboring node. The probability that the two nodes are involved in transmission may be obtained from a normal probability distribution function [21]. In a WSN, if we take any two nodes, the modeling makes use of a probability function as weighting function to indicate whether the two nodes are involved in transmission or not. So the normal distribution weighting *φ*(*x*) may be defined as

(4) $$\begin{array}{c} \varphi \left(x\right)=\frac{1}{\sqrt{2\pi }}e^{-x^{2}/2}. \end{array}$$

The probability that a phenomenon is sensed and transmitted to the base station that involves two nodes (sensor node and base station node) is obtained by substituting a value of *x* = 2 in the above equation. If the nodes are linearly arranged (as in most of the cases), there are two possibilities of phenomenon being sensed and transmitted, represented by *c*1 and *c*2. Hence, the probability of sensing a data and transmitting to the base station with only two nodes involved is given by

(5) $$\begin{array}{c} f\left(x\right)=\varphi \left(x\right)\left|c1+\varphi \left(x\right)\right|c2=2\times \varphi \left(x\right)=2\times \frac{1}{\sqrt{2\pi }}e^{-x^{2}/2}. \end{array}$$

Hence, the total power (*P*_*T*_) is given by

(6) PT=(et+er+ep)×f(x)×navg=(et+er+ep)×{2×12πe−x2/2}×navg.

Substituting this in (2), NLT_max⁡_ is calculated as

(7) $$\begin{array}{c} {\text{NLT}}_{\max}=\frac{E_{i}}{\left(e_{t}+e_{r}+e_{p}\right)\times \left\{2\times \left(1/\sqrt{2\pi }\right)e^{-x^{2}/2}\right\}\times n_{\text{avg}}}. \end{array}$$

This value of NLT_max⁡_ is further influenced by how the nodes are placed (topology or node placement pattern) over the entire sensing field. If the nodes are assumed to be placed all over the sensing field uniformly, a pattern similar to Pattern 1 is obtained. So, Pattern 1 is assumed as the reference pattern and the corresponding NLT_max⁡_ is given in (7). The NLT_max⁡_ of other patterns or topologies may be modeled as follows:

(8) $$\begin{array}{c} {\text{NLT}}_{\max\left(\text{topo}\right)}={\text{NLT}}_{\max}\times \gamma^{\text{topo}}, \end{array}$$

where *γ* ^topo^ is a cost factor depending on the topology.

The main differences between each node placement pattern or topology are

- the number of nodes,
- the pattern of node placement,
- the density of nodes.

Hence, the cost factor *γ* ^topo^ may be modeled based on the number of nodes in each pattern with respect to the reference pattern. Again by heuristic this cost factor is modeled as

(9) $$\begin{array}{c} \gamma^{\text{topo}}=\sqrt{\frac{D_{p}}{D_{\text{ALL}}}}, \end{array}$$

where *D*_*p*_ is the density in the pattern for which NLT_max⁡_ is to be calculated and *D*_ALL_ is the density in the reference pattern, that is, ALL pattern. Density = number of nodes/total area.

Hence the general form of NLT_max⁡_ is given by

(10) NLTmax⁡=Ei(et+er+ep)∗{2∗(1/2π  )e−x2/2}∗navg  ∗γtopo

or

(11) NLTmax⁡=Ei(et+er+ep)∗{2∗(1/2π)e−x2/2}∗navg  ∗DpDALL.

This NLT model is used for evaluating the maximum NLT of the different node distribution patterns. This maximum NLT is evaluated for the prescribed values of initial energy, energy consumed for transmission, reception and processing, number of nodes of each pattern, and so forth. This theoretical value may be compared with the value of NLT obtained by conducting the experiments.

## 5. Node Distribution Patterns for WSN Interconnected with Fixed Network

**Figure 4:**
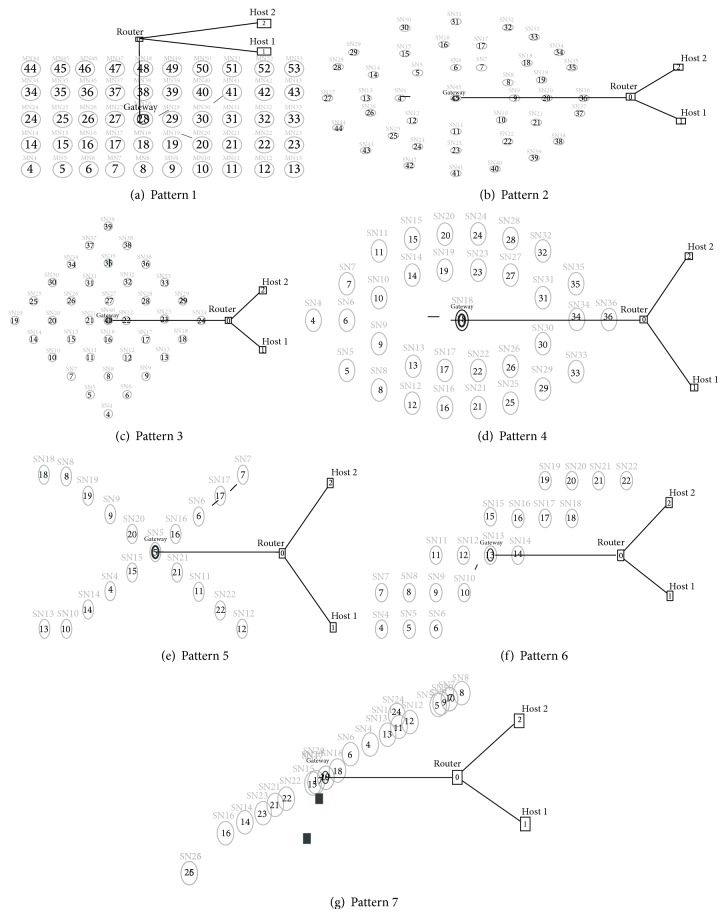
Various WSN pattern simulations.

Placement of sensor nodes plays a very important role in sensor networks [8, 22, 23]. Efficiency of signal detection depends on the position of the sensor nodes. Hence this paper analyses seven different distribution patterns of placing the nodes.  Figure 4 shows the seven node distribution patterns simulated. Pattern 1 is shown in Figure 4(a). Pattern 1 is a matrix of sensor nodes with nodes placed in every cross section. This pattern has redundant nodes but is more efficient to detect the physical phenomenon to be sensed within the entire area.

A mobile node is used to simulate the occurrence of a physical phenomenon that is to be sensed by the WSN. Similarly, Pattern 2 to Pattern 7 are simulated as shown in Figures 4(b) to 4(g). These seven sensor node placement patterns are used to evaluate their performance. Patterns 1 to 7 simulated are aimed at providing placement patterns that may be used for several applications such as structural monitoring of building, street light control, earth quake detection, and habitat monitoring. By using different protocols such as AODV, DSDV, LEACH, and HETRA, energy efficiency and NLT analysis are made. For the analysis, each routing protocol is simulated with five different TCP variants. The TCP variants used for simulation are TCP/Tahoe, TCP/Reno, MIMD-Poly, PIPD-Poly, and TCP/EXP. Simulation is performed with the packets transmitted from the phenomenon node. This transmission of packet simulates that the phenomenon is occurring at the place of phenomenon node and the received packet by sensor node simulates that as the sensed data. This simulated sensed data is now transmitted to the base station node through the other intermediate nodes. The simulation scenario generated is available in a nam file. When this file is executed in a nam editor, the simulated network is displayed. 

### 5.1. Simulation Program

Simulation experiments are conducted in ns 2 using the seven node distribution patterns (see Box 1). Coverage of a sensor node is evaluated by conducting a simulation experiment with only two wireless nodes. In this simulation experiment the height of the antenna and the transmission power are varied to find the minimum height required and the optimum power for covering a diagonal distance of 80 m is found for each pattern. By using various parameters like channel type, propagation mode, network interface type, mac type, interface queue type, antenna, interface queue length, routing protocol, and antenna height the wireless sensor network interconnected with fixed TCP/IP network is simulated with the seven node distribution patterns discussed earlier in this section.

Protocols such as AODV, DSDV, LEACH, and HETRA are used for analyzing the energy efficiency and NLT of WSN connected with fixed wired network. Each of these routing protocols is simulated with five different TCP variants such as TCP/Tahoe, TCP/Reno, MIMD-Poly, PIPD-Poly, and TCP/EXP.

Scenario of the simulation experiments conducted is available in a nam file. When this file is executed in a nam editor, the simulated network is displayed as shown in Figure 4. All the wireless nodes and nodes in the fixed network are allotted with hierarchical addressing. The hierarchical addressing in ns 2 uses the following format:

Domain_address. Cluster_address. Node_address.

The nodes are divided into two domains: one containing wireless nodes including the base station node and the other containing nodes of fixed network. The interconnection between WSN and fixed network is through the gateway node. The data sensed by the sensor nodes are sent to the gateway node through the intermediate sensor nodes. These data received by the gateway node from the various sensor nodes are collected and locally processed. The information processed is sent to the sink node through the fixed TCP/IP network, by sending them first to the router node and then to the required sink node. The query-response makes use of the hierarchical addresses while sending a query to the sensor node as well as while transmitting the response to the sink node.

The program segment used to define the hierarchical addressing space is given below:

 AddrParams set domain_num_ 2,
 AddrParams set cluster_num_ $\left\{\begin{array}{cc} 1 & 1 \end{array}\right\}$,
 AddrParams set nodes_num_ $\left\{\begin{array}{cc} 3 & 51 \end{array}\right\}$.

The entire network nodes are grouped into two domains. Each domain consists of a single cluster. Cluster 0 in domain 0 consists of nodes of fixed network, that is, one router and two host nodes. Cluster 0 in domain 1 consists of all the wireless sensor nodes and gateway node. Gateway node acts as the interconnected node between the wireless and fixed network. Based on these assignments of domain and cluster, each node is allotted with the IP address as given in the program segment.

With these address allocations, a wireless sensor network interconnected with fixed network topology is simulated. Similarly the hierarchical addressing for the router and host nodes is allocated for remaining six node distribution patterns.

## 6. Simulation Results 

**Figure 5:**
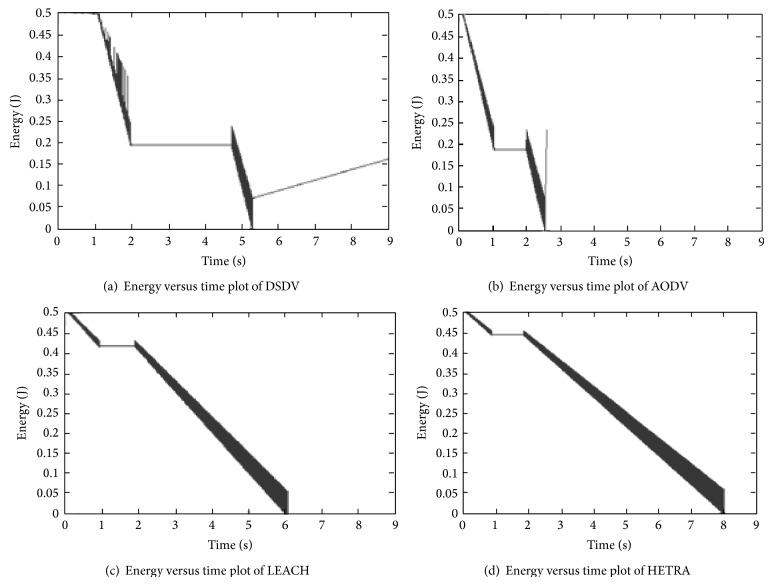
Remaining energy of ALL pattern sensor nodes with different routing algorithms and TCP/EXP.

HETRA is implemented as a new WSN routing protocol in ns 2. Fixed routing mechanism is used in this HETRA. The logical HET is constructed every 2 seconds in the simulation experiments conducted. But, this HET refreshing time in real time may in the order of minutes. Trace files are generated during the simulation of different patterns with routing and congestion control algorithms. The content of this file is used to evaluate the NLT. The evaluated NLT for various simulations are shown in Figure 5. The figure shows that the initial energy of 0.5 Joules reduces to zero and this duration is the network lifetime of each simulated network pattern.

Assuming the TCP/EXP congestion control, Figure 5 gives the values of NLT for DSDV, AODV, LEACH, and HETRA routing protocols using the node distribution Pattern1. In the same way, the NLT is calculated for the remaining other six node distribution patterns. The results clearly indicate that the combination of HETRA with TCP/EXP has longer network lifetime compared with the other routing algorithms for all the node distribution patterns.

**Figure 6:**
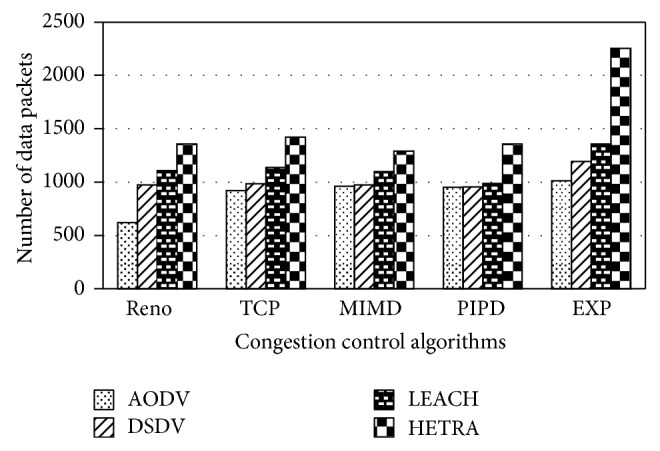
Number of data packets for Pattern 1.

**Figure 7:**
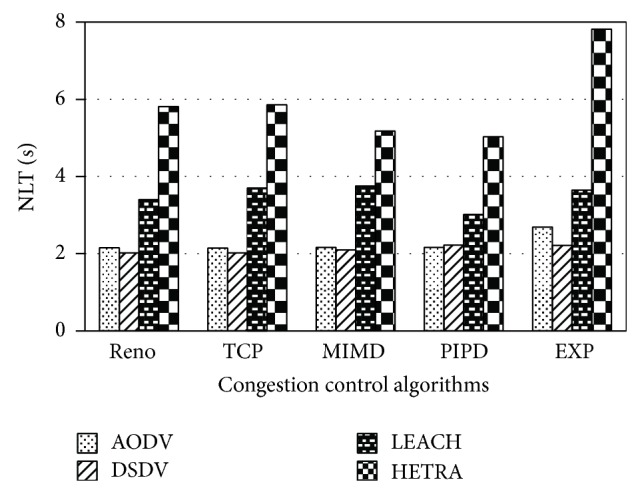
NLT for Pattern 1.

**Figure 8:**
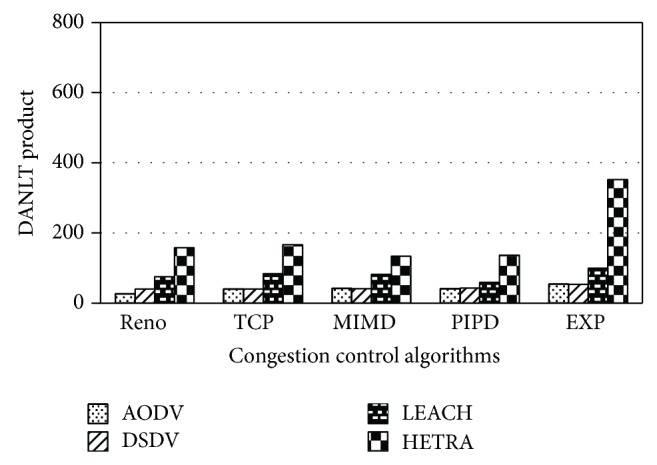
DANLT product for Pattern 1.

**Figure 9:**
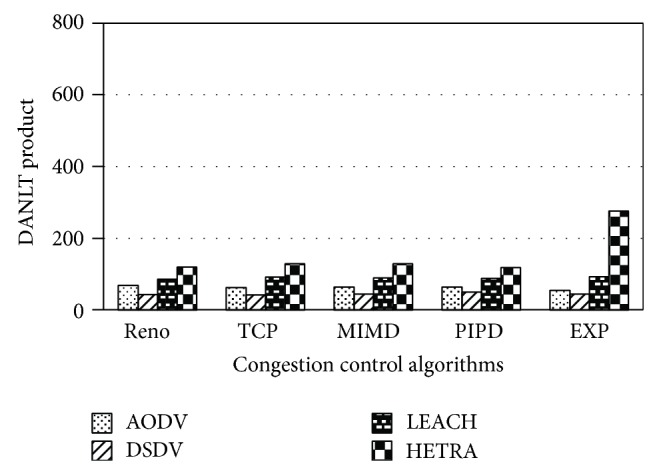
DANLT product for Pattern 2.

**Figure 10:**
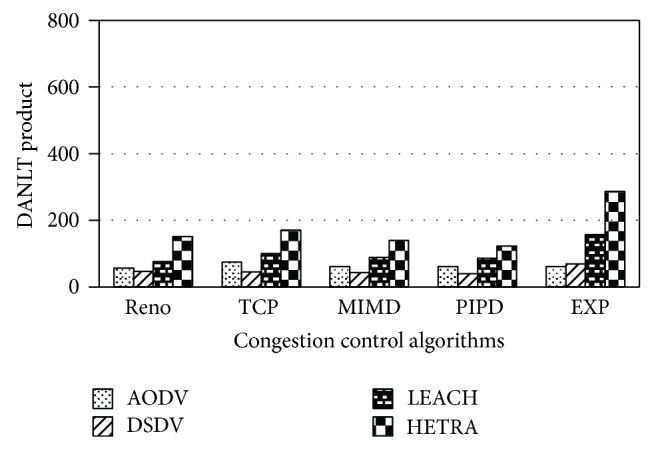
DANLT product for Pattern 3.

**Figure 11:**
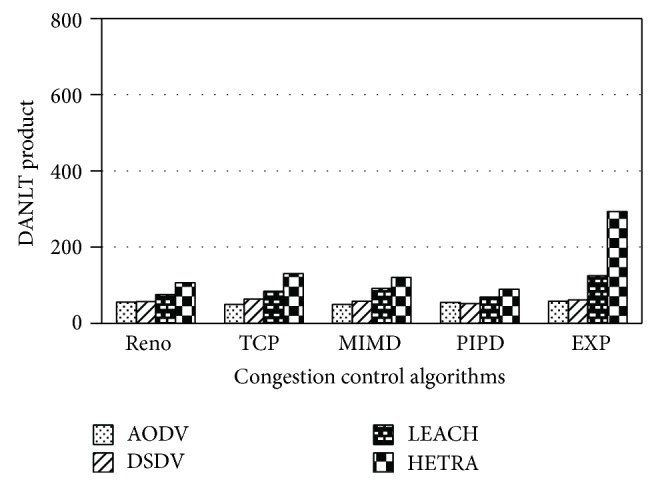
DANLT product for Pattern 4.

**Figure 12:**
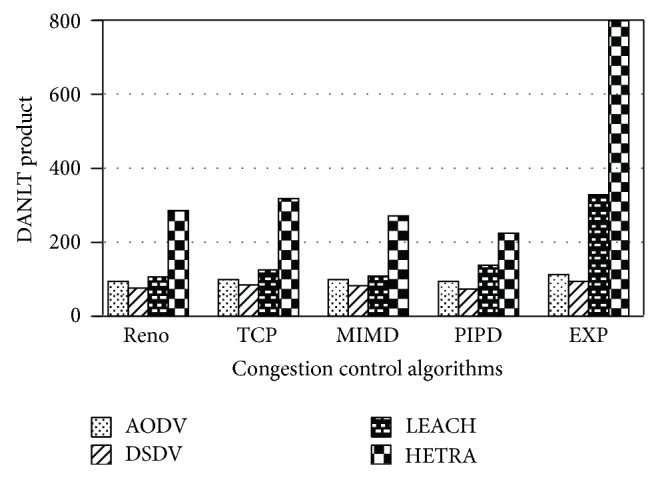
DANLT product for Pattern 5.

**Figure 13:**
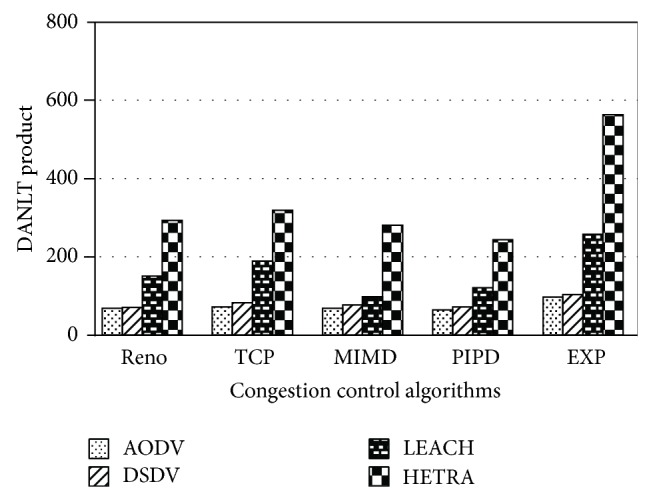
DANLT product for Pattern 6.

**Figure 14:**
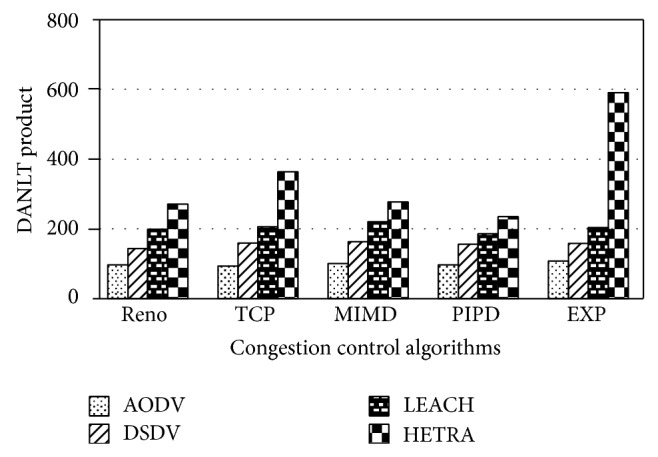
DANLT product for Pattern 7.

Figures 6 to 8 show the values of number of data packets delivered, NLT, and DANLT product for Pattern1. Similarly the number of data packets delivered, NLT, and DANLT product are calculated for the remaining other six node distribution patterns for various congestion control algorithms with various routing algorithms. Figures 9 to 14 show the DANLT product for the remaining other six node distribution patterns. This shows that the combination of HETRA with TCP/EXP performs better for all the node distribution patterns compared with other routing and congestion control algorithms. This combination has higher energy efficiency, throughput, and number of data packets delivered. This shows that HETRA/EXP combination improved the overall efficiency of the sensor network.

In order to combine three parameters: NLT, data packets, and number of nodes a common parameter DANLT product is devised. Average network lifetime (ANLT) is evaluated first. From the simulation experiments conducted for the different patterns, ANLT is calculated.

 Average network lifetime per node = network lifetime (NLT)/number of nodes.
 DANLT product = number of data packets delivered × average network lifetime (ANLT).

**Figure 15:**
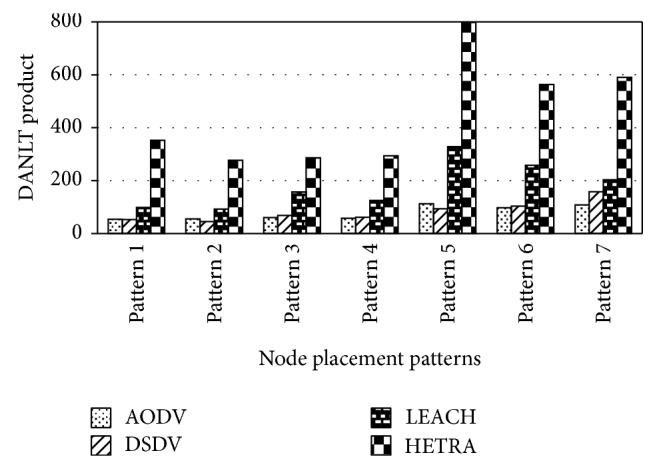
DANLT product for various routing algorithms with TCP/EXP congestion control.

Figure 15 shows the values of DANLT product for seven node distribution patterns for various routing algorithms of AODV, DSDV, LEACH, and HETRA with TCP/EXP as the congestion control algorithm.

The result clearly indicates that the combination of HETRA with TCP/EXP performs better for all the node distribution patterns. As the number of nodes in Pattern 5 is less compared with other patterns and the energy efficiency is comparable with other patterns, Pattern 5 is the most energy efficient pattern in terms of energy efficiency, number of data packets, and throughput among the seven fixed node distribution patterns assumed.

## 7. Conclusion and Future Research

This paper is aimed at designing an energy efficient routing algorithms for WSN. Two algorithms are proposed. One is for routing sensed packets using hierarchical energy tree (HET) and is called HETRA. The other is to control the number of data packets dropped due to congestion and is called exponential congestion control algorithm (TCP/EXP). The improvement of energy efficiency is further attempted by using seven fixed node distribution patterns for WSN. The WSNs in these patterns are connected to a fixed network. The proposed algorithms HETRA and TCP/EXP are implemented in ns 2. The performances of the proposed algorithms are compared with the existing routing and congestion control algorithms. This paper is aimed at analyzing the NLT of WSN for seven node distribution patterns with the combination of routing and congestion control algorithms. This paper is assumed to have seven patterns, four routing algorithms, AODV, DSDV, LEACH, and HETRA, and five TCP congestion control algorithms, TCP, Reno, MIMD, PIPD, and TCP/EXP.

HETRA and TCP/EXP algorithms are implemented in ns 2 and simulation experiments are performed. The results of these simulation experiments are analyzed. Parameters such as number of data packets received, NLT, ANLT, and DANLT product are evaluated using the trace files generated during these simulation experiments. The NLT of each simulation with TCP/EXP congestion control algorithm, four routing protocols in Pattern 1 is shown in Figure 5. These parameters are compared in Figures 6, 7, 8, 9, 10, 11, 12, 13, 14, and 15. The results clearly show that the combination of HETRA with TCP/EXP maximizes longer network lifetime for all the node distribution patterns compared with other routing and congestion control algorithms. Among these patterns Pattern 5 performs most efficiently in terms of the energy efficiency, network lifetime, number of data packets delivered, and throughput.

The future work will be on forming the efficient pattern by placing the nodes randomly and readjusting the positions by evaluating their DANLT product. Optimization techniques may also be used for maximizing the DANLT product by adjusting the node positions.

## Figures and Tables

**Box 1:**
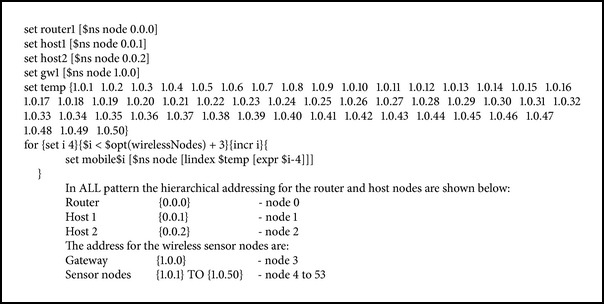
Simulation Program.

**Algorithm 1:**
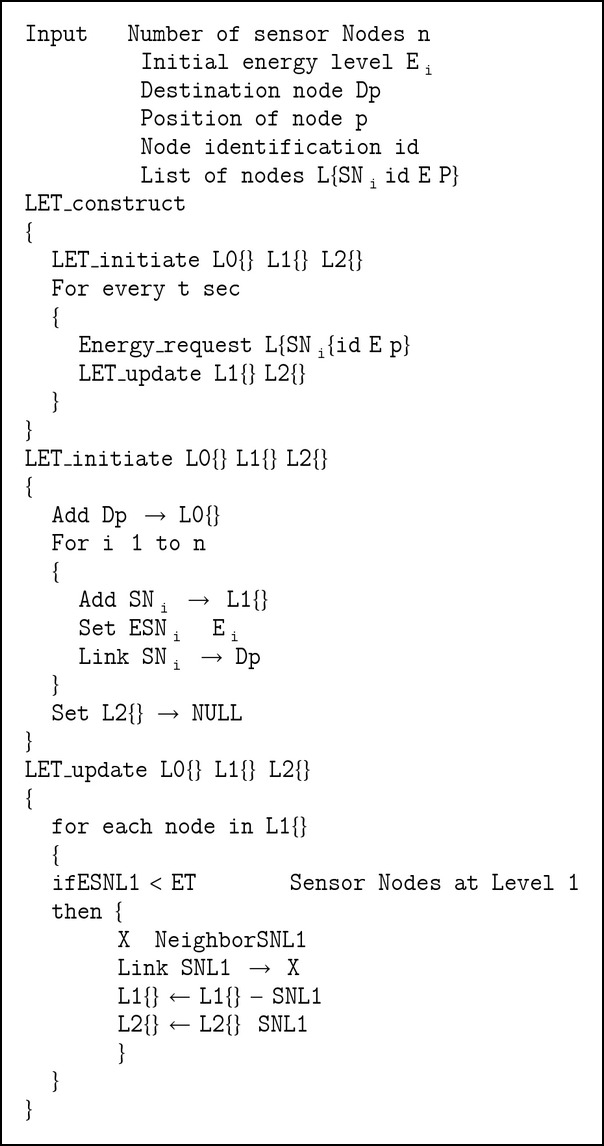
HET Construction Algorithm.
